# Screening and functional analysis of the differential peptides from the placenta of patients with healthy pregnancy and preeclampsia using placental peptidome

**DOI:** 10.3389/fgene.2022.1014836

**Published:** 2022-12-01

**Authors:** Tingting Chen, Zhongxiao Zhang, Qin Lu, Jun Ma

**Affiliations:** ^1^ Department of Gynaecology and Obstetrics, Tongren Hospital, Shanghai Jiaotong University School of Medicine, Shanghai, China; ^2^ Hongqiao International Institute of Medicine, Tongren Hospital, Shanghai Jiao Tong University School of Medicine, Shanghai, China; ^3^ Department of General Practitioners, Tongren Hospital, Shanghai Jiao Tong University School of Medicine, Shanghai, China

**Keywords:** peptides, placenta, preeclampsia, peptidomics, TGF-β/Smad signaling pathway

## Abstract

Molecular peptides play an extensive range of functions in the human body. However, no previous study has performed placental peptidome profiling. In the present study, 3,941 peptides from human placental tissues were identified using peptidomics. Compared to healthy pregnant women, there were 87 and 129 differentially expressed peptides (DEPs) in the mild and severe preeclampsia groups, respectively. In the mild PE group, 55 and 34 DEPs had high and low expressions, respectively. In comparison, in the severe PE group, 82 and 47 DEPs had high and low expressions, respectively. Functional analysis of the precursor proteins of DEPs by gene ontology suggested that they are primarily involved in focal adhesion, extracellular matrix-receptor interaction, tight junction, and extracellular matrix. Network analysis using ingenuity pathway analysis software showed that the precursor proteins of DEPs were primarily related to the transforming growth factor-β (TGF-β)/Smad signaling pathway. Further molecular docking experiments showed that the AASAKKKNKKGKTISL peptide (placenta-derived peptide, PDP) derived from the precursor protein IF4B could bind to TGF-β1. Therefore, our preliminary results suggest that the actions of PDP may be mediated through the TGF-β1/Smad signaling pathway. Our results demonstrate that the placental bioactive peptides may regulate the placental function during PE progression.

## Introduction

Preeclampsia (PE) is characterized by hypertension and proteinuria after 20 weeks of gestation (Rana et al., 2019). PE is the leading cause of maternal and fetal diseases and death. PE affects 5%–8% of pregnant women in developing countries (Ramos et al., 2017). The progression of PE to eclampsia is associated with severe complications, leading to adverse maternal and fetal outcomes. There is no effective treatment for eclampsia and its definitive treatment is the termination of pregnancy (Phipps et al., 2019). Premature birth and even abortion caused by premature termination of pregnancy are associated with significant burden on the family and society. Therefore, it is necessary to develop additional treatments for PE. PE is prevented by the use of aspirin (Atallah et al., 2017); however, studies indicate that 30%–40% of high-risk pregnant women develop PE despite the use of aspirin (Walsh et al., 2020). Due to the late onset of symptoms and limited treatment options for PE, biomarkers are being developed for earlier detection and treatment (Ma’ayeh and Costantine 2020).

In the previous decade, several studies have focused on the development of diagnostic markers of PE (Stepan et al., 2020). Placental ischemia triggers the pathogenesis of PE and stimulates the release of placental anti-angiogenic factors, such as soluble fms-like tyrosine kinase-1 (sFlt-1) and soluble endoglin (Herraiz et al., 2018). Given the importance of Flt-1 in pregnancy, the *Flt-1* gene may serve as a genetic marker for PE susceptibility. Placental protein 13 (PP13) levels are also elevated in PE (Wu et al., 2021). In addition, blood pressure during pregnancy is positively related to the level of pregnancy-associated plasma protein A (PAPP-A) (Serra et al., 2020). However, it is still unknown whether these markers are effective for screening for PE.

In recent years, endogenous peptides, a class of naturally occurring bioactive molecules, were identified. Several studies have reported their wide tissue distribution and strong biological effects (Czuba et al., 2018). As important regulators of cardiovascular homeostasis, endogenous peptides enter the blood by autocrine or paracrine pathways, participate in blood pressure regulation, and protect the body and reduce injury in pathological states. Human β-defensin 2 selectively acts on calcium-activated potassium (BK) channels to reduce the diastolic and systolic blood pressure and blood flow velocity; because of these effects, β-defensin 2 is expected to be useful as a new drug treatment of hypertension (Liu et al., 2013). Glucagon-like peptide 1 (GLP-1) improves vascular endothelial cell injury and treats diabetic atherosclerosis by regulating the NF-Kb signal pathway (Ma et al., 2021). So far, no study has examined the difference in placental peptidome between normotensive pregnant women and those with mild and severe PE. PE is resolved with the delivery of the placenta (Shaheen et al., 2020), indicating that the placenta is the primary contributor to the pathogenesis of PE (Wang et al., 2019). Therefore, it is essential to screen placental bioactive peptides.

In this study, placental tissues from women with a healthy pregnancy, mild PE, and severe PE were used for peptidomics. The purpose was to screen the differentially expressed peptides (DEPs) of PE and identify the functional peptides that may be useful for the prevention and treatment of PE.

## Materials and methods

### Sample collection

This study was approved by the ethical research committee of Shanghai Tongren Hospital (2018-060-01). The median age of the patients was 31 years (range: 28–33). The study participants were pregnant singleton women, without previous organic diseases or hypertensive disorders before the pregnancy. The patients fulfilled the criteria for mild or severe PE proposed by the guidelines of the Chinese Society of Obstetrics and Gynecology. PE refers to high blood pressure (systolic blood pressure ≥ 140 mmHg or diastolic blood pressure ≥ 90 mmHg on two consecutive occasions) occurring after 20 weeks of pregnancy in women with preterm babies. The control group included healthy women with no signs of hypertension or proteinuria. The placental tissues from three patients with severe PE (*n* = 3), mild PE (*n* = 3), and healthy, gestational age-matched pregnant women (*n* = 3) were collected from the department of Obstetrics and Gynecology at Shanghai Tongren Hospital and stored at −80°C until analysis.

### Preparation of samples for LC-MS/MS

Tris-HCl was added at a volume ratio of 1:3, boiled for 10 min, and then cooled in an ice bath. Next, a 100-Hz ultrasonic wave was administered for 5 s with an interval of 5 s followed by 2 min of ultrasonic wave. The sample tube was filled with glacial acetic acid at a final concentration of 1 M, followed by 2 min of vortex oscillation. The supernatant was collected and frozen. A solution of 80% acetone was added, vortexed, shaken, and added to the water bath. Then, the solution was subjected to ultrasonic waves for 2 min at 4°C and 20,000 rpm. Next, the solution was subjected to high-speed centrifugation for 30 min. The supernatant was collected, transferred to a clean centrifugal tube, and freeze-dried. Next, 200 μl of 0.1% TFA solution was added to the solution and desalting was performed on a C18 column using a sample loading of 80 μg. The sample was freeze-dried and subsequently analyzed by LC-MS.

### LC-MS/MS analysis

Mass spectrometry was performed using Q Exactive with C18 (3 μm, 150 mm × 5 µm). Mass spectrometry: positive ion detection mode, first-order resolution of 70,000, AGC set to 3e6, and scanning range of 300–1,400 m/z. Top 20 ions were selected for MS/MS analysis. The secondary resolution was 17,500, AGC was set to 5e4, and the isolation window was 3 m/z. For liquid chromatography, the chromatographic column was C18 (3 μm, and 250 mm × 75 µm). The mobile phase A was 0.1% formic acid; mobile phase B was acetonitrile with 0.1% formic acid. The flow rate was 300 nl/min and the injection volume was 6 µl. The mobile phase gradient was 0–8 min for 6%–10% B, 8–60 min for 8%–30% B; 60–79 min for 30%–42% B; 79–80 min for 42%–95% B; 80–85 min for 95% B; 85–86 min for 95%–6% B; and 86–90 min for 6% B. The total running time was 90 min.

### Bioinformatics

MAXQUANT (Bruker Daltonics) was used to process the data. Mascot (version 2.5.1) and Swissprot *Homo sapiens* database were used for the database search, considering the Oxidation (M) Acetyl (Protein N-term) as variable modifications. The search was conducted to identify non-specifc peptides with at least six amino acids. Other parameters were set as default. There were 3,941 peptide matches. The peak intensity values were normalized by summing the peaks in each sample. The Mann-Whitney U test was used to estimate the differences in peptide representation among the groups. The data were uploaded to the website (http://bioinformatics.psb.ugent.be/webtools/venn/) to construct a Venn diagram. Volcano plots and heatmaps were constructed using the R package. Using the R package, principal component analysis (PCA) was performed to create a classification model: control, mild PE, and severe PE.

### Function analyses

Gene ontology (GO) was used to analyze the properties and functions of precursor proteins of DEPs in terms of biological processes (BPs), molecular functions (MFs), and cellular components (CCs). The KEGG pathway database was used to identify the most important signaling pathways and metabolic pathways related to the precursor proteins. The interaction analysis of precursor proteins of DEPs was carried out using IPA software. The interaction network of precursor proteins was constructed, and the enrichment analysis of related signal pathways was carried out.

### Molecular docking

The peptide (AASAKKKNKKGKTISL) structure was obtained from Maestro 11.9. Then the peptide was imported to the Chem3D software for optimize and minimize the energy by using the MM2 module, and saved as a sdf file as a ligand molecule for molecular docking. The TGF-β1 (PDB ID: 6P7J) protein structure was downloaded from the RCSB database (https://www.rcsb.org/). The protein structure is processed on Maestro11.9 platform. The protein is processed with Schrodinger’s Protein Preparation Wizard to remove crystal water, add missing hydrogen atoms, repair missing bond information, and repair missing peptide segments. Finally, the protein was then minimized using the OPLS3 force field. The processing and optimization of molecular docking was completed by Glide module in Schrödinger Maestro software (Schrödinger, New York, NY, United States). While performing the docking of this peptide to TGF-β1, five poses of the ligand, were produced by the SP mode of Glide. The ligand interaction diagram module of Glide was used to analyze ligand-protein interactions.

## Results

### Identification of differentially expressed peptides

**FIGURE 1:**
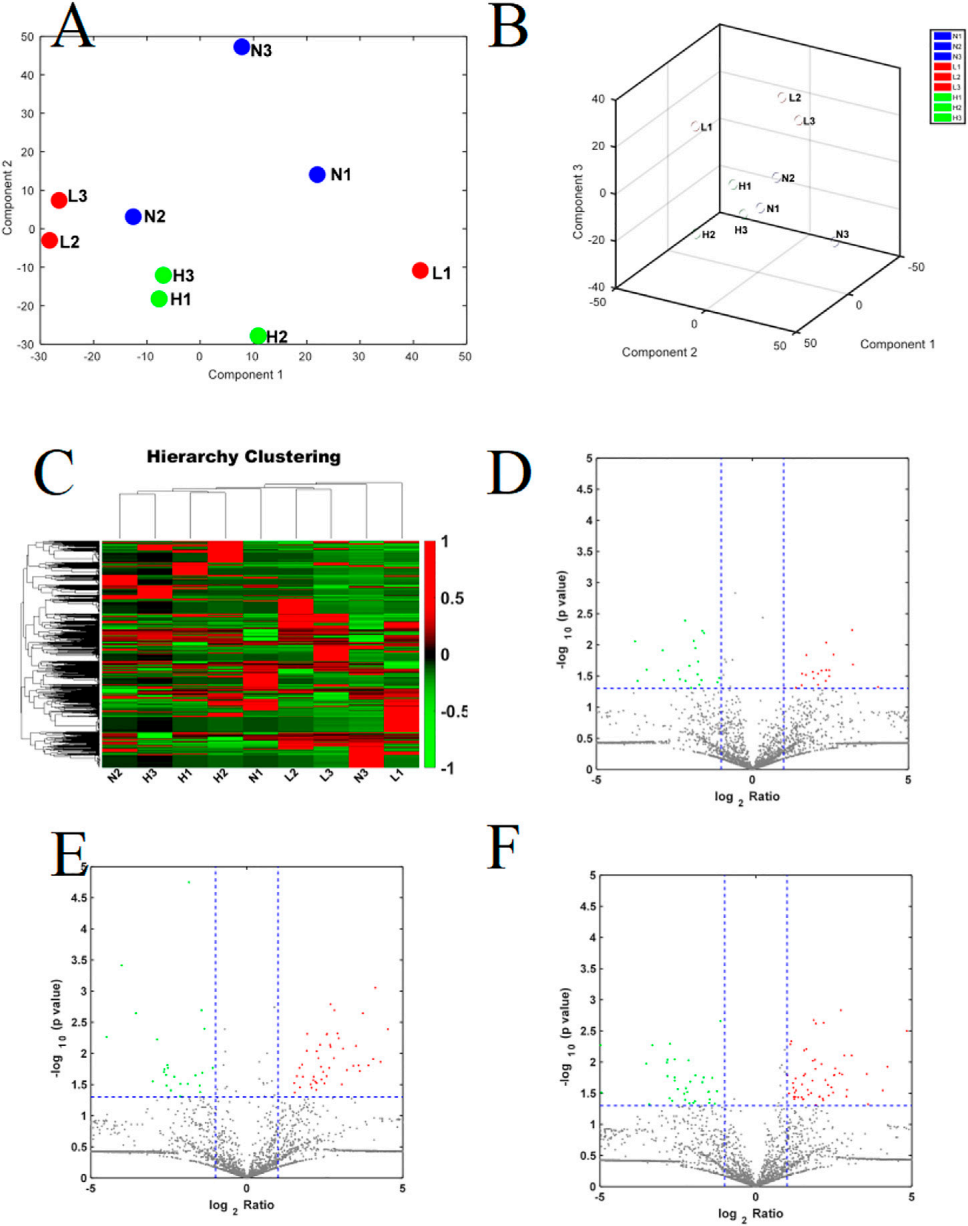
**(A,B)** The PCA score plot of severe PE/control groups, mild PE/control groups; L, mild PE. H, severe PE; N, control group; **(C)** Heatmap of DEPs in the comparison of severe PE, mild PE and control groups; L, mild PE. H, severe PE; N, control group. **(D)** Volcano plot of DEPs in the comparison of mild PE/control group; **(E)** Volcano plot of DEPs in the comparison of severe PE/control group; **(F)** Volcano plot of DEPs in the comparison of severe PE/mild PE; each point in the figure represents a peptide. The red region is the upregulated peptide, and the green region is the downregulated peptide.

**FIGURE 2:**
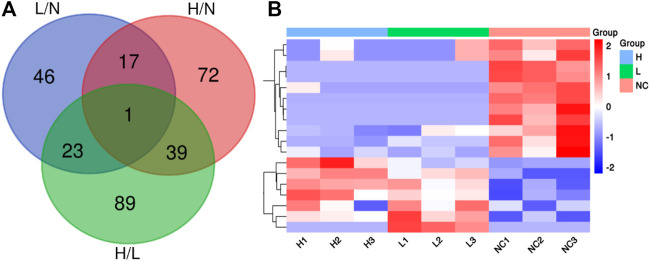
**(A)** Venn diagram showing the overlap of DEPs in the comparison of severe PE/control groups, mild PE/control group and severe PE/mild PE; **(B)** Heatmap analysis of 18 DEPs both in the comparison of severe PE/control group and mild PE/control group. L, mild PE; H, severe PE; N, control group.

We identified 3,941 peptides. These data were used for multivariate analysis. The three groups were separated by PCA analysis, as shown in Figures 1A,B. Compared to healthy pregnant women, 51 peptides were increased, whereas 36 were decreased, in patients with mild PE (fold change > 2, *p* < 0.05; Supplementary Table S1). Additionally, 82 peptides were increased and 47 peptides were decreased in patients with severe PE (fold change > 2, *p* < 0.05; Supplementary Table S2). Compared to healthy pregnant women, 78 peptides were upregulated and 74 peptides were downregulated in severe PE patients (fold change > 2, *p* < 0.05; Supplementary Table S3). A heatmap and Volcano map were used to display the DEGs (Figures 1C–F). Compared to healthy pregnant women, 18 peptides were significantly changed both in mild and severe PE groups, as shown in the Venn plot and heatmap (Figures 2A,B). Only one peptide (GHFTEEDKATI) was significantly altered in the abovementioned three comparisons (Table 1). GO analyses revealed that many peptides were related to the negative regulation of the apoptotic process, translational initiation, translation, and other extracellular matrices (Supplementary Figure S1).

**TABLE 1:** DEPs both in the comparison of mild PE/control and severe PE/control.

Sequence	Entry name	Log ratio (L/N)	*p*-value (L/N)	Log ratio (H/N)	*p*-value (H/N)	Log ratio (H/L)	*p*-value (H/L)
AASAKKKNKKGKTISL	IF4B	−11.10	8.53E-06	−10.69	8.54E-06	0.42	0.15
ADIQTERAYQKQPTIFQNKKRVLL	RS11	−1.12	4.00E-02	−1.35	4.02E-03	−0.23	0.69
AHVDDMPNALSA	HBA	2.60	1.44E-02	3.51	7.54E-03	0.90	0.06
ANRGPAYGLSREVQQKI	TAGL2	−6.76	2.09E-03	−6.34	2.12E-03	0.42	0.15
DQEAIQDLWQWRKSL	NPM	2.37	2.55E-02	2.21	2.86E-02	−0.16	0.54
DVFLGMFLYEYAR	ALBU	−2.16	4.09E-03	−3.55	2.25E-03	−1.39	0.07
GHFTEEDKATI	HBG1	1.94	2.71E-02	4.12	8.84E-04	2.17	0.00
IENEEQEYVQTVK	ANXA1	2.22	2.60E-02	2.95	5.68E-03	0.73	0.05
IENPGFEASPPAQGIPEAKVRHPLS	VISTA	−7.73	1.21E-02	−7.31	1.22E-02	0.42	0.15
LDPITGRSRGFGFVLF	HNRPD	−7.96	1.47E-03	−2.53	1.72E-02	5.43	0.37
QMGTNRGASQAGMTGYGMPRQIL	TAGL2	−2.14	3.80E-02	−2.55	1.53E-02	−0.41	0.85
SGDAAIVDMVPGKPM	EF1A1	−6.27	6.52E-04	−5.85	6.63E-04	0.42	0.15
SGGKYVDSEGHL	CAV1	6.07	1.33E-03	0.25	4.43E-01	−5.82	0.00
SLSPFYLRPPSFLRA	CRYAB	−1.96	4.91E-02	−2.13	3.10E-02	−0.17	0.94
SRNGMVLKPHFHKDWQRRVATWF	RL13	−3.76	8.76E-03	−1.90	3.07E-02	1.86	0.27
STMAFKQMEQISQFLQAAERY	TAGL2	−6.14	9.99E-03	−5.72	1.02E-02	0.42	0.15
VETRDGQVINETSQ	VIME	1.72	3.02E-02	2.55	4.94E-03	0.84	0.01
VSESSDVLPK	K2C8	1.57	4.42E-02	1.17	4.16E-01	−0.40	0.61

L/N, represent the comparison of mild PE/normal; H/N, represent the comparison of severe PE/normal; H/L, represent the comparison of severe PE/mild PE.

### Bioinformatics

**FIGURE 3:**
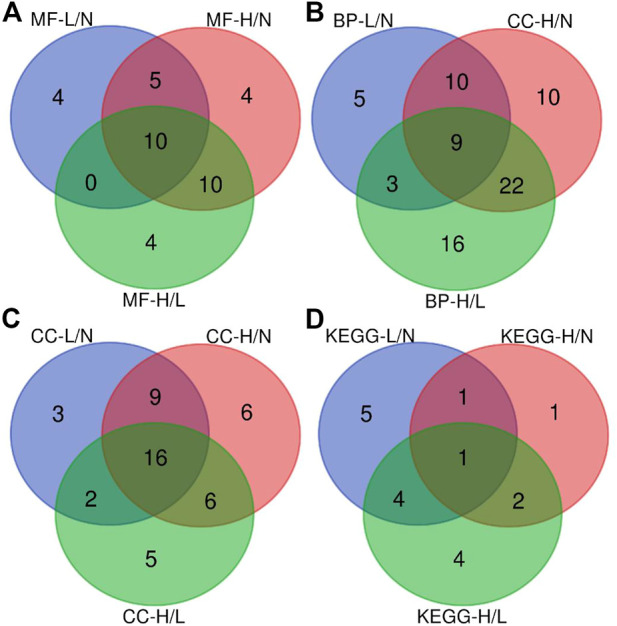
Venn diagram showing the overlap of the enriched biological pathways identified using GO and KEGG pathway databases. **(A)**, Venn diagram of the overlap of significant regulated molecular function categories; **(B)**, Venn diagram of the overlap of significant regulated biological process categories; **(C)**, Venn diagram of the overlap of significant regulated cellular component categories; **(D)**, Venn diagram of the overlap of significant regulated KEGG pathway categories. L/N, mild PE/control; H/N, severe PE/control group; H/L, severe PE/mild PE.

**FIGURE 4:**
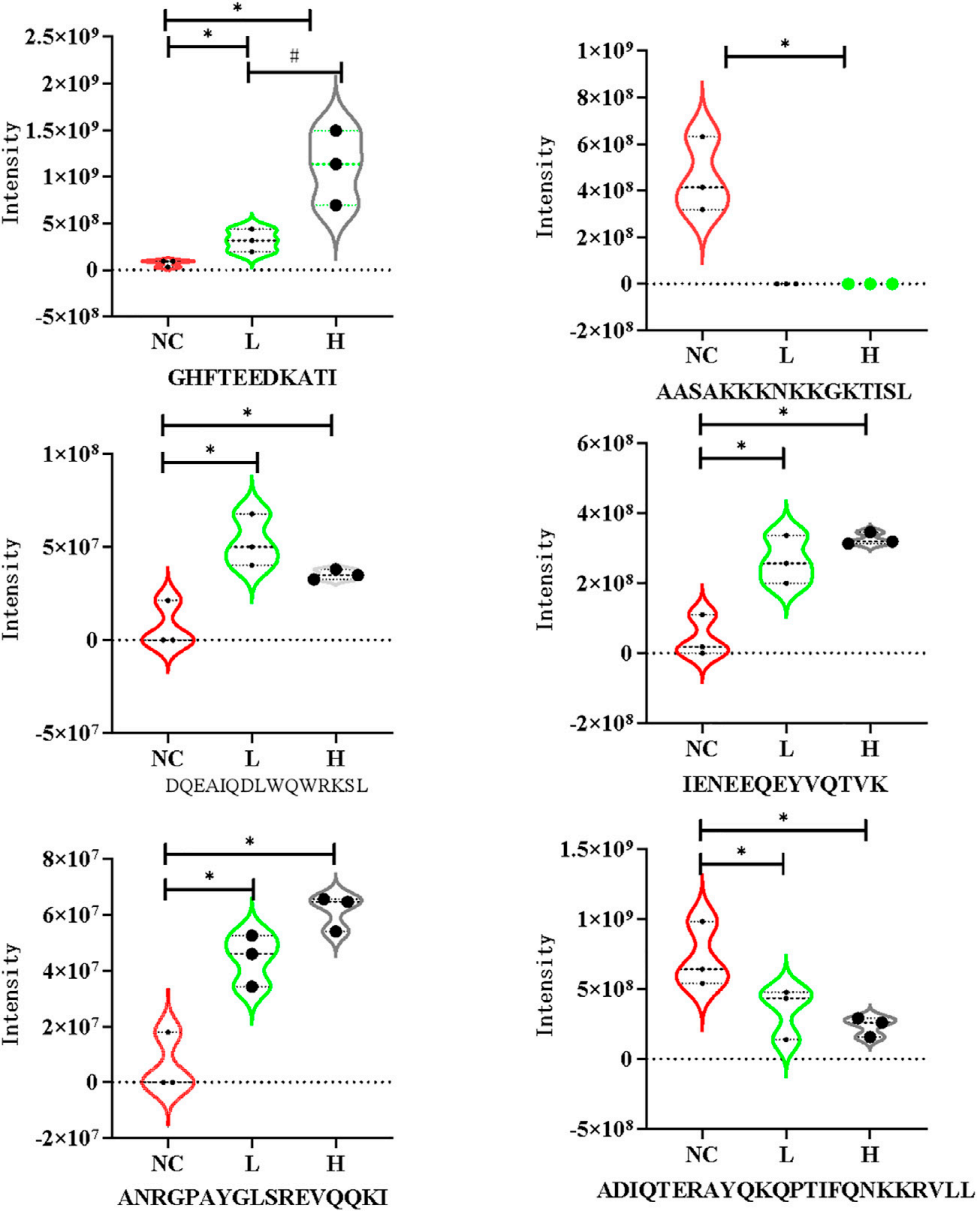
Violin plots of DEPs in the mild PE, severe PE and control group. L, mild PE; H, severe PE; NC, control group. *, represent *p* < 0.05 compared to the control group #, represent *p* < 0.05 compared to the mild PE group.

The CCs related to the DEPs included extracellular exosome, focal adhesion, extracellular space, extracellular region, and extracellular matrix. The precursor proteins of DEPs were enriched in oxidative phosphorylation, arrhythmogenic right ventricular cardiomyopathy, oxidative phosphorylation, hypertrophic cardiomyopathy, dilated cardiomyopathy, and tight junction, as well as with the occurrence of PE (Figure 3). The contents of the related PEPs in these pathways among different groups are shown in Figure 4.

**FIGURE 5:**
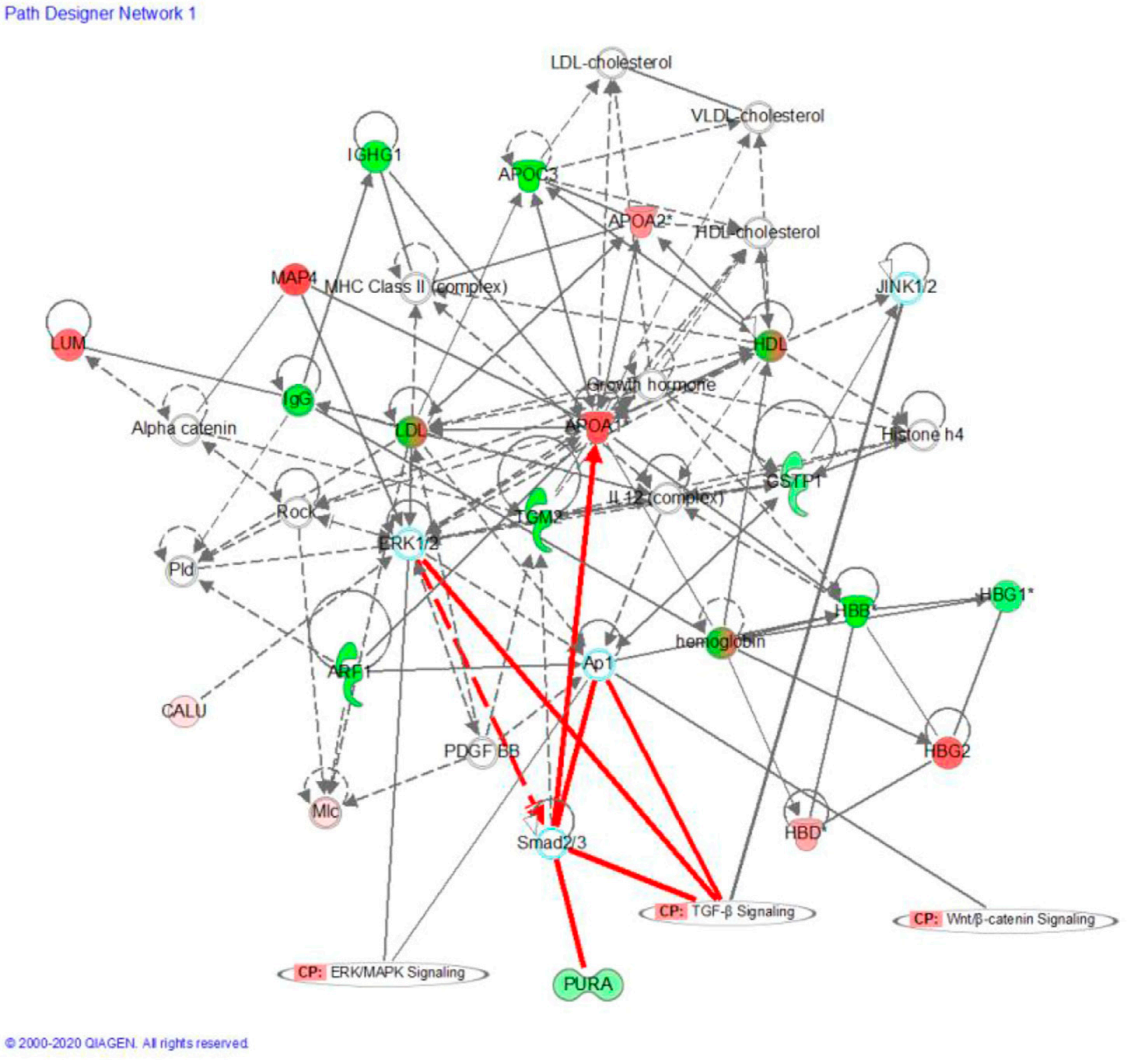
Precursor protein of DEPs analysis by Ingenuity Pathway Analysis (IPA); Red and blue dots represent the significantly up- and downregulated DEPs related Precursor Proteins, respectively; “CP” is an abbreviation for “canonical pathway,” which represents a signaling pathway related to the highly linked molecules. Indirect relationships are represented by dotted lines and direct relationships by solid lines.

**FIGURE 6:**
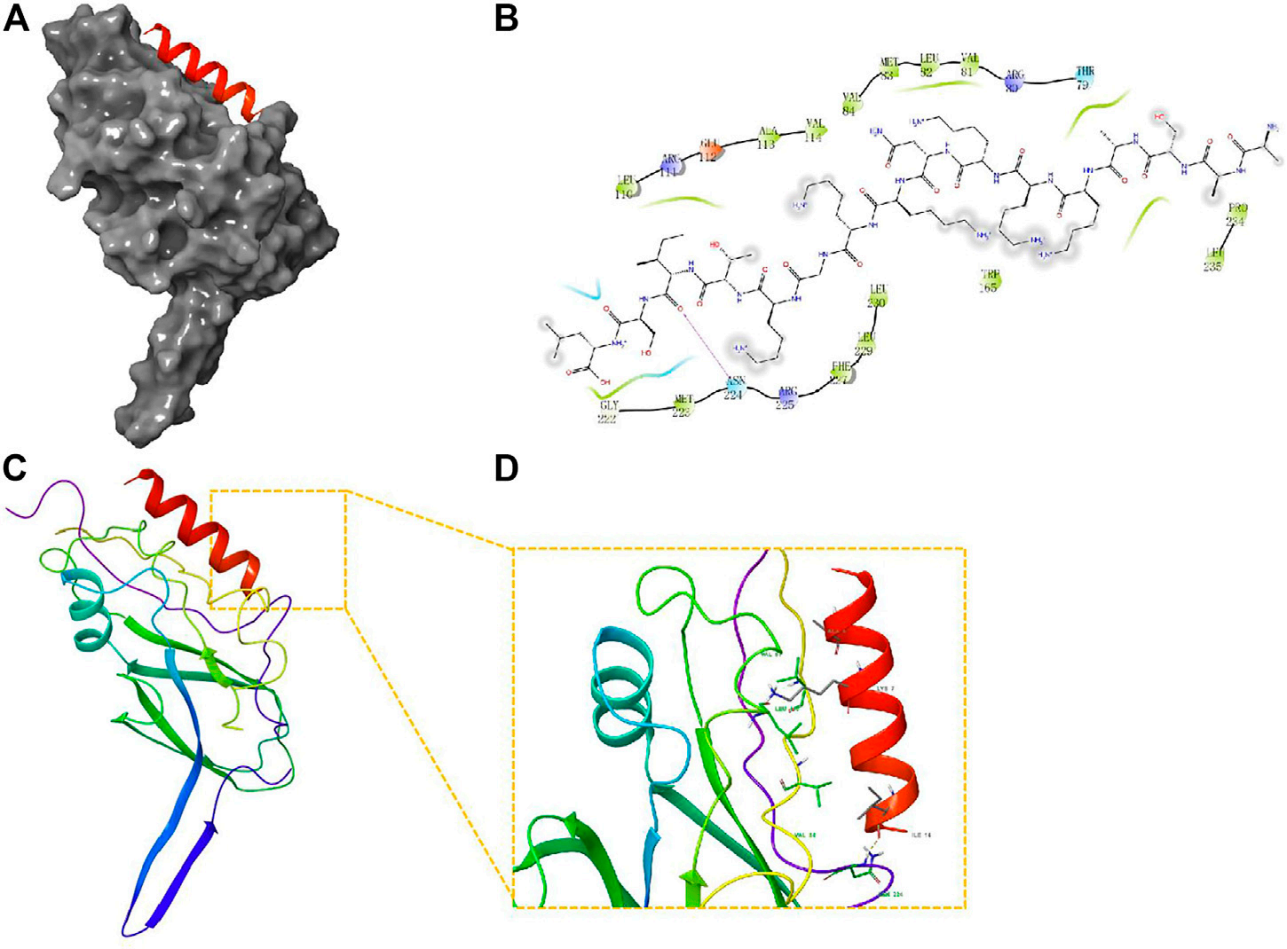
Molecular docking study of PDP to TGF-β1. **(A)** The 3D structure of docked molecule binding to TGF-β1; **(B)**, Binding sites of PDP and TGF-β1, the binding site amino acid residue ASN (224); **(C,D)**; Close-up view of binding site of PDP docked with TGF-β.

Network analysis using IPA showed that the changes in precursor proteins of DEPs were mainly related to the TGF-β/Smad signaling pathway (Figure 5). Further molecular docking experiments showed that the AASAKKKNKKGKTISL peptide (PDP) derived from the precursor protein IF4B could bind to TGF-β1, suggesting that TGF-β1 may be the target protein of PDP (Figure 6). The docking score of PDP was −28.57 kcal/mol. PDP could be docked with the molecule of TGF-β1 Serine residue at the position of ASN 224.

## Discussion

Peptidomics is used to study the peptides in biological samples, which are important because the soluble molecules can provide valuable information about an individual’s physical status, changes in behavior, and severity of illness (Foreman et al., 2021). Several studies have evaluated the urine and serum peptides in pregnant women with PE (Law et al., 2015; Dai et al., 2017; Qian et al., 2017; Kononikhin et al., 2020; Starodubtseva et al., 2020). However, no previous study has investigated placental peptides. PE is resolved with the delivery of the placenta (Shaheen et al., 2020); thus, the placenta is the primary contributor to the pathogenesis of PE (Wang et al., 2019). There is evidence that placenta and placental peptides may play an important role in PE. Thus, it is worth investigating the peptidome profiles in the placenta of PE patients.

In our study, several biologically active peptides derived from 480 precursor proteins were altered during PE development. Compared to a previous study, a greater number of peptides was identified in the placenta than in the serum and urine of PE patients (Kononikhin et al., 2020). This may be due to the release of a greater number of endogenous peptides from the placenta, indicating that the greater number of peptides may provide additional options for PE treatment. Compared to healthy pregnant women, those with mild PE showed upregulation of 51 peptides and downregulation of 36 peptides. In patients with severe PE, 82 peptides were upregulated and 47 peptides were downregulated. Of these DEPs, 18 peptides were altered between the severe and mild PE compared to the control groups. The panel peptides were derived from six protein precursors: ANXA1, CAV1, IF4B, RS11, HBA, and TAGL2.

Notably, IENEEQEYVQTVK was increased in severe and mild PE patients; this protein was derived from the protein Annexin A1 (ANXA1). A previous study revealed that the level of the anti-inflammatory protein ANXA1 was also increased in early PE (Perucci et al., 2015; Feng et al., 2018). In our study, the level of the peptide IENEEQEYVQTVK derived from AnxA1 was higher in mild and severe PE compared to healthy pregnant women. The increased AnxA1-derived peptide level may counter the inflammatory response in PE patients. No previous study has evaluated the AnxA1-derived peptide in the placenta from PE and normotensive patients. A prospective study is required to explore the use of AnxA1-derived peptide as a PE biomarker.

The pathways significantly associated with the precursor protein of DEPs primarily included “focal adhesion”, “tight junction”, and “extracellular matrix”. Evidence indicates that PE develops due to the presence of the placenta (Lim et al., 2015; Mohammadpour-Gharehbagh et al., 2018). Placenta maintains the transport between the fetus and mother (Jena et al., 2020; Melchiorre et al., 2022). For fetal development and placental embedding, extravillous trophoblasts must invade the decidua of the mother, while it can be disrupted in conditions related to pregnancy, such as PE (Ma et al., 2020). Abnormal placentation caused by impaired trophoblast function causes pregnancy-associated syndromes, such as PE (Schoots et al., 2018). There is growing evidence to suggest that ECM interactions play a significant role in trophoblast proliferation and differentiation (Wong et al., 2018). In trophoblasts, ECM thickness affects the production of mRNA and proteins related to fusion. Abnormal expression of placental ECM components is associated with abnormal migration/invasion of human trophoblast cells (Romanowicz and Galewska 2011; Ma et al., 2020; Liu et al., 2021; Parameshwar et al., 2021). Consequently, PE may develop due to ECM changes.

To uncover the potential efficacy of peptides in PE, network analysis was performed using IPA, which showed that the changes in the precursor proteins of DEPs were mainly related to the TGF-β/Smad signaling pathway. Further molecular docking experiments showed that AASAKKKNKKGKTISL peptide (PDP) derived from the precursor protein IF4B could bind to TGF-β1, suggesting that TGF-β1 may be the target protein of PDP. Thus, targeting the TGF-β/Smad signal pathway is an effective strategy for the treatment of PE. Ligand binding activates the receptors, and TGF-β phosphorylates Smad2 and Smad3 (Xu et al., 2016). Activated Smad2 and Smad3 bind to Smad4 to regulate the transcription of several genes that contribute to trophoblast invasion and proliferation (Brkić et al., 2020). PDP is an endogenous peptide that was identified by peptidomics screening; no functional report has evaluated PDP so far. PDP may act as a potential bioactive peptide to improve PE by acting on the TGF-β signal pathway. PDP has the advantages of low molecular weight, high stability, good lipophilicity, and easy entry into cells and nuclei. PDP may be a new method for the clinical treatment of PE. Therefore, future studies are required to study its function and mechanism.

## Conclusion

In the present study, we identified DEPs from the placenta of patients with mild and severe PE. Functional analysis suggested that the precursor proteins of DEPs were mainly involved in PE progression. Further molecular docking experiments showed that PDP peptides derived from the precursor protein IF4B could bind to TGF-β1, suggesting that PDP may play its biological function by affecting the TGF-β1/Smad signaling pathway. Our results suggest some strategies for the discovery of active peptides, which may be helpful for the diagnosis and treatment of PE.

## Data Availability

The datasets presented in this study can be found in online repositories. The names of the repository/repositories and accession number(s) can be found in the article/Supplementary Material.
