# Supplementary material for: Screening and functional analysis of the differential peptides from the placenta of patients with healthy pregnancy and preeclampsia using placental peptidome
Source: Front Genet. 2022 Dec 1;13:1014836. doi: 10.3389/fgene.2022.1014836 (PMC9751626; doi:10.3389/fgene.2022.1014836)
Supplement: Supplementary file 1 [file Table1.pdf]

Supplemental Material

Screening and functional discovery of differential peptides in placenta of normal and preeclampsia

Tingting Chen[1, #], Zhongxiao Zhang[3, #], Qin Lu[1, *], Jun Ma[1, 2, *]

1 Department of Gynaecology and Obstetrics, Tongren Hospital, Shanghai Jiaotong University School of Medicine, 1111 XianXia Road, Shanghai 200336, China.

2 Department of General Practitioners, Tongren Hospital, Shanghai Jiao Tong University School of Medicine, 1111 XianXia Road, Shanghai, 200336, China.

3 Hongqiao International Institute of Medicine, Tongren Hospital, Shanghai Jiao Tong University School of Medicine, 720 XianXia Road, Shanghai 200336, China.

# Both authors contributed equally to this work.

* Corresponding author, Jun Ma, Department of General Practitioners, Tongren Hospital, Shanghai Jiao Tong University School of Medicine, 1111 XianXia Road, Shanghai, 200336, China.

E-mail address: majun@shtrhospital.com.

Qin Lu, Department of Gynaecology and Obstetrics, Tongren Hospital, Shanghai Jiaotong University School of Medicine, 1111 XianXia Road, Shanghai 200336, China. Email address:LQ1982@shtrhospital.com.

Supplemental Material Table S1. DEPs in the comparison of mild PE/normal.L/N, represent the the comparison of mild PE/normal.

Sequence	Entry	LogRatio(L/N)	p Value(L/N)
SDKPDMAEIEK	P62328	9.01	5.31E-03
SSFLPWIRT	P08311	8.87	5.52E-03
SRPVRNRKVVDYSQF	Q9H1E3	8.84	3.13E-02
HDNEETFLKKYLYEI	P02768	8.70	4.92E-04
VVRHQLLKT	P15954	8.54	3.14E-02
SSARPGGLGSSSLYGLG	P08729	8.15	2.48E-03
AEDMETKIKNY	P14854	8.10	1.49E-04
VGSQATDFGEALVRHDEF	O43852	7.78	3.77E-03
DDMPNALSALSDLHAHKLRVDPV	P69905	7.47	6.05E-03
NSFVNDIFERIAGEAS	P06899	7.37	2.67E-02
SGGNYRDNYDN	P98179	7.26	3.71E-02
DYDALDVANKIGII	P62750	7.25	1.52E-02
RDLAKDITSDTSGDFRNALL	P04083	7.14	2.97E-03
ADKPDMGEIASFDK	P63313	7.09	2.71E-02
ENRLQNMEVTDA	P49591	7.00	1.63E-02
NDEELNKLLGKV	P0C0S8	7.00	5.42E-03
DPELCAVTFVDRPVIG	P16278	6.76	2.50E-02
SVVTGPAPSQEAGTKARFPLRDA	P21980	6.72	1.86E-02
RETNLDSLPLVDT	P08670	6.68	3.98E-04
SEKAKPALEDLRQGLLPVLESFKV	P02647	6.51	2.24E-04
ELFEQLGEYKF	P02768	6.46	2.88E-03
AQQAADKYLYVDKN	P35579	6.35	4.22E-04
NYQPPTVVPGGDLAKV	Q9BQE3	6.33	3.76E-02
AEDMETKIKNYK	P14854	6.10	6.69E-04
SGGKYVDSEGHL	Q03135	6.07	1.33E-03
SFLPWIRTTMR	P08311	6.05	1.56E-03
LENVIRDAVTY	P62805	6.05	3.18E-02
GSGGGSFGDNLVTRSY	P02545	5.82	2.97E-03
HELQEKLSPL	P02647	5.82	1.46E-02
TEAPLNPKANREKMTQI	P60709	5.69	1.17E-03
DIAVDGEPLGRVS	P62937	5.61	4.15E-03
VDDMPNALSALSDLHAHKLRVDPV	P69905	5.41	7.47E-03
NKELDPIQKLFVDKIREY	P18859	5.37	5.97E-03
DIRPEIHENYRING	O75348	5.20	3.02E-02
LEEYTKKLNTQ	P02647	4.03	4.75E-02
DDDIAALVVDNGSGMCK	P60709	3.23	2.06E-02
TRDGQVINETSQ	P08670	3.19	5.80E-03
AHVDDMPNALSA	P69905	2.60	1.44E-02
VYKVLKQVHPDTGI	P06899	2.47	2.54E-02

YTKKVPQVSTPTLVEV	P02768	2.46	3.17E-02
DQEAIQDLWQWRKSL	P06748	2.37	2.55E-02
AGFAGDDAPRAVFPSIVGRPRHQGV	P60709	2.37	9.25E-03
SSGVSEIRHTA	P04792	2.35	3.81E-02
SWNSGALTSGVHTFPA	P01857	2.32	4.99E-02
IENEEQEYVQTVK	P04083	2.22	2.60E-02
SDKPDMAEIEKFDKSK	P62328	2.11	4.38E-02
ALPRAQEGLRPG	P08311	2.10	2.90E-02
MNSFVNDIFERIAGEAS	P06899	1.97	3.43E-02
GHFTEEDKATI	P69891	1.94	2.71E-02
ELRVAPEEHPVLL	P60709	1.73	1.45E-02
VETRDGQVINETSQ	P08670	1.72	3.02E-02
TLDGGFIYEAGLAPYKLRPV	P02788	1.60	2.89E-02
VSESSDVLPK	P05787	1.57	4.42E-02
DLAGRDLTDYLMK	P60709	1.39	4.89E-02
GEYKFQNALLV	P02768	1.02	4.99E-02
MDGIVPDIAVGTK	P26599	-1.06	3.38E-02
ADIQTERAYQKQPTIFQNKKRVLL	P62280	-1.12	4.00E-02
SQAYSSSQRVSSY	P17661	-1.55	6.48E-03
VDYHAANQSYQYGP	O43707	-1.61	5.94E-03
QKVVAGVAN	P02042	-1.64	3.70E-02
SGNFGGSRNMGGP	P22626	-1.70	4.39E-02
SQAYSSSQRVSS	P17661	-1.74	1.84E-02
ASSDIQVKELEKRASGQ	P16949	-1.76	3.00E-02
EERYKTGKNKWFFQKLRF	P61353	-1.82	1.13E-02
VIGLQMGTNRGASQAG	P37802	-1.88	8.65E-03
SLSPFYLRPPSFLRA	P02511	-1.96	4.91E-02
MYATDSRGHSP	Q9UHN6	-2.01	2.18E-02
QMGTNRGASQAGMTGYGMPRQIL	P37802	-2.14	3.80E-02
DVFLGMFLYEYAR	P02768	-2.16	4.09E-03
SSRSYTSGPGSRISSSSFSRVGSSN	P05787	-2.32	3.58E-02
VLAHHFGKEFTPPVQA	P68871	-2.38	2.59E-02
SQAYSSSQRVS	P17661	-2.83	3.67E-02
DNLKGTFATL	P68871	-2.87	1.22E-02
AGGHKLGLGLEFQA	P21796	-3.39	2.53E-02
VDYHAANQSYQYGPSSAGN	O43707	-3.68	3.76E-02
SRNGMVLKPHFHKDWQRRVATWF	P26373	-3.76	8.76E-03
AASGSGMAQKTWELANN	Q92905	-6.10	5.64E-03
STMAFKQMEQISQFLQAAERY	P37802	-6.14	9.99E-03
SQAYSSSQR	P17661	-6.17	1.52E-02
SGDAAIVDMVPGKPM	P68104	-6.27	6.52E-04
ANRGPAYGLSREVQQKI	P37802	-6.76	2.09E-03
MERFVVTAPPARN	Q9UFW8	-7.36	8.61E-03
MEVMNLMEQPIKVTE	P14923	-7.50	7.40E-03

IENPGFEASPPAQGIPEAKVRHPLS	Q9H7M9	-7.73	1.21E-02
LDPITGRSRGFGFVLF	Q14103	-7.96	1.47E-03
NVLKVTKAAGTKKQFQKF	P62280	-8.72	1.31E-02
AASAKKKNKKGKTISL	P23588	-11.10	8.53E-06

Supplemental Material Table S2. DEPs in the comparison of severe PE/ normal. H/N, represent the the comparison of severe PE/ normal.

Sequence	Entry	LogRatio(H/N)	p Value(H/N)
TERRVPFSLLRGPSWDPFRDWY	P04792	10.18	4.17E-04
VVRHQLLKT	P15954	9.20	4.60E-03
VHLTPEEK	P02042	8.94	1.05E-04
TQEKNPLPSKETIEQEKQAGES	P62328	8.24	6.39E-03
VLSPANTQPTPLP	Q8NEZ4	8.05	4.88E-03
SVELEEALPV	Q92522	7.84	9.94E-03
SESSSKSSQPL	P17096	7.84	3.01E-03
FLSFPTTKT	P69905	7.72	1.00E-03
DIRKDLYANT	P60709	7.70	9.01E-09
YSVEFSEEPFGVIVRRQLD	P10253	7.63	3.68E-02
IYTRNTKGGDAPAAGEDA	P62851	7.44	1.12E-02
KGTFATLSELHCDKL	P68871	7.41	2.89E-03
ADMQNLVERLERAVGR	Q01518	7.29	3.63E-03
SGGNYRDNYDN	P98179	7.29	2.41E-02
ERMFLSFPTTKTYF	P69905	7.28	4.43E-02
EQQNKILLAELEQL	P08670	7.17	1.73E-03
FDDVVGETVGKTD	P27816	6.98	1.04E-03
KDSGRDYVSQFEGSALGKQLNL	P02647	6.71	1.20E-03
ADALTNAVAHVD	P69905	6.45	6.69E-03
NTDWGDNGFFK	P07858	6.43	1.20E-03
EQEYVQTVK	P04083	6.23	3.57E-02
NFRGGLGGG	P05787	6.11	4.25E-02
FGDNLVTRSY	P02545	6.09	1.24E-02
VTGVASALSSRY	P69892	6.04	1.69E-02
GAVEAISDR	P07858	6.03	3.17E-02
PHFDLSHGSAQV	P69905	5.88	4.45E-03
MDDFERRRELR	Q05682	5.87	8.32E-03
GESDDSILRL	P63220	5.86	8.35E-03
HVDDMPNALSALSDLHAH	P69905	5.79	9.37E-03
ARLPSGLPVSLLTL	P51884	5.74	2.26E-02
DQDEIQRLPGLA	P10619	5.46	1.42E-03
SLAALKKALAA	P10412	5.37	4.32E-03
FLRAQERAAES	P46777	5.29	2.89E-07
SFLRAQERAAES	P46777	5.15	8.25E-04

VLGGVDVTGPHLY	Q99436	5.05	1.09E-02
IEQNTKSPLFMGKVVNPTQK	P01009	4.52	4.11E-03
FVELGTQPAT	P02652	4.28	1.37E-02
GHFTEEDKATI	P69891	4.12	8.84E-04
VKPVKVSAPRVGGKR	P83731	4.05	1.23E-02
FLSFPTTKTYFPHFDLSHG	P69905	3.90	1.56E-02
NLQTVNVDEN	P62899	3.72	2.27E-03
ASLDKFLASVSTVLTSKYR	P69905	3.62	1.92E-02
WGKVGAHAGEYGAEALERMFLSF	P69905	3.59	1.54E-02
AHVDDMPNALSA	P69905	3.51	7.54E-03
LVVYPWTQRF	P02042	3.41	1.59E-02
IVDGKVVSETNDTKV	P05783	3.21	1.86E-02
RVDPVNFKLLSHCLLVTL	P69905	3.09	8.38E-03
ADALTNAVAHVDDMPNALSAL	P69905	3.05	3.13E-02
IENEEQEYVQTVK	P04083	2.95	5.68E-03
STVHEILCKLSLEGDH	P07355	2.91	4.81E-03
LMIEQNTKSPLFMGKVVNPTQK	P01009	2.81	2.04E-03
AWGKVGAHAGEYGAEAL	P69905	2.79	1.18E-02
MIEQNTKSPLFMGKVVNPTQK	P01009	2.68	1.62E-03
ENEEQEYVQTVK	P04083	2.68	7.86E-03
KVSFLSALEEYTKKLNTQ	P02647	2.67	7.23E-03
HVDPENFKLLGNVLVT	P69891	2.65	1.71E-02
LDSLPLVDTHSK	P08670	2.58	4.88E-03
SFPTTKTYFPHFDL	P69905	2.56	2.32E-02
VETRDGQVINETSQ	P08670	2.55	4.94E-03
HVDDMPNALSAL	P69905	2.49	3.01E-02
ETRDGQVINETSQ	P08670	2.45	1.96E-02
AAHLPAEFTPAVHASLDKF	P69905	2.39	2.61E-02
NLQEAEEWYKSK	P08670	2.38	1.63E-02
FVELGTQPATQ	P02652	2.36	7.27E-03
LSALEEYTKKLNTQ	P02647	2.30	1.19E-02
AWGKVGAHAGEYGAEALERMFL	P69905	2.25	9.13E-03
HVDDMPNALSALSDLHAHKL	P69905	2.24	3.00E-02
PWTQRFFDSFGNLSS	P69891	2.23	3.88E-02
DQEAIQDLWQWRKSL	P06748	2.21	2.86E-02
HLPAEFTPAVHA	P69905	2.14	2.36E-02
DALTNAVAH	P69905	2.14	2.67E-02
ALTNAVAHVDDMPN	P69905	2.07	3.54E-02
VDDMPNALSALSDLHAHKL	P69905	2.04	3.13E-02
HVDPENFKLLGNVLVTVL	P69891	1.93	4.87E-03
SALSDLHAHKL	P69905	1.92	9.09E-03
HAGEYGAEALERM	P69905	1.79	2.34E-02
AWGKVGAHAGEYGAEALERMFLSF	P69905	1.77	1.50E-02
AIHFGKEFTPEVQA	P69891	1.70	3.50E-02

TTEVGSVSEVKKDSSQ	O43491	1.69	1.72E-02
GNLSSASAIMGNPKVK	P69891	1.61	2.31E-02
AWGKVGAHAGEYGAEALERM	P69905	1.54	4.26E-02
HAGEYGAEAL	P69905	1.52	3.00E-02
AQFVNWIDSIIQR	P08246	-1.09	1.71E-02
ADIQTERAYQKQPTIFQNKKRVLL	P62280	-1.35	4.02E-03
NLPINGNGKQ	P09211	-1.43	2.05E-02
AYHSFLVEPI	O15143	-1.45	2.03E-03
VHLTPEEKSAVTALW	P68871	-1.50	4.17E-02
LPINGNGKQ	P09211	-1.50	3.26E-02
AGAPTVSLPELRSLL	Q8NFU3	-1.64	2.25E-02
SLLALYKGKKERPRS	P62266	-1.85	1.78E-05
SRNGMVLKPHFHKDWQRRVATWF	P26373	-1.90	3.07E-02
SGGKYVDSEGHLYTVP	Q03135	-2.06	4.93E-02
NRPTQPLKGRTVRASF	P00915	-2.12	4.88E-02
SLSPFYLRPPSFLRA	P02511	-2.13	3.10E-02
RLLLPGELAKHAV	P06899	-2.32	2.34E-02
LEQQNKILL	P08670	-2.43	3.91E-02
LDPITGRSRGFGFVLF	Q14103	-2.53	1.72E-02
QMGTNRGASQAGMTGYGMPRQIL	P37802	-2.55	1.53E-02
SYTSGPGSRISSSSFSRVGSSN	P05787	-2.57	3.26E-02
MVGMGQKDSYVGDEAQSKRGILTL	P60709	-2.58	2.22E-02
RGGLGGGYGGASGMGGITAV	P05787	-2.65	1.79E-02
EGKVLPGVDALSNI	P00558	-2.65	1.97E-02
GLQMGTNRGASQAGMTGYGMPRQIL	P37802	-2.88	5.96E-03
SSGVSEIRHTADRWRV	P04792	-3.02	2.80E-02
DVFLGMFLYEYAR	P02768	-3.55	2.25E-03
IVGGRRARPHAWPFMV	P08246	-4.01	3.89E-04
SSVAVLTQESFAEHR	Q00341	-4.49	5.46E-03
AGEYGAEALERMFL	P69905	-5.33	1.15E-03
YNEATGGKYVPRAIL	P07437	-5.56	2.87E-02
NEATGGKYVPRAVL	P68371	-5.63	2.60E-04
STMAFKQMEQISQFLQAAERY	P37802	-5.72	1.02E-02
SGDAAIVDMVPGKPM	P68104	-5.85	6.63E-04
DMLQGIIGAKHS	P27105	-5.97	7.78E-03
SPPPPPPPGGAWEAVRVPR	Q13219	-6.19	2.39E-05
SLGSVQAPSYGARPV	P05783	-6.23	2.46E-02
AAGVEAAAEVAATEIK	P52272	-6.31	2.84E-03
ANRGPAYGLSREVQQKI	P37802	-6.34	2.12E-03
LAAAGYDVEKNNSRIKLGLK	P10412	-6.52	1.86E-02
GLQMGTNRGASQAG	P37802	-6.97	2.64E-03
TLSQPKIVKWDRDM	P61769	-7.04	3.79E-03
LDWARERLEQQVPVNQVF	P39023	-7.07	3.15E-02
GDLSTPDAVMGNPKVK	P68871	-7.07	2.17E-02

AAKVFESIGKFG	P35232	-7.28	1.96E-03
GTNRGASQAGMTGYGMP	P37802	-7.30	4.66E-02
IENPGFEASPPAQGIPEAKVRHPLS	Q9H7M9	-7.31	1.22E-02
IDTTSKFGHGRFQTM	P39023	-7.72	4.96E-03
TRVSSFLPWIRTTMR	P08311	-7.73	4.95E-02
VHLTPEEKSAVTALWG	P68871	-8.17	2.62E-02
AASAKKKNKKGKTISL	P23588	-10.69	8.54E-06

Supplemental Material Table S3. DEPs in the comparison of severe PE/ mild PE. H/L, represent the the comparison of severe PE/ mild PE.

Sequence	Entry	LogRatio(H/L)	p Value(H/L)
FVELGTQPAT	P02652	8.34	1.07E-02
VLSPANTQPTPLP	Q8NEZ4	8.22	4.88E-03
PWTQRFFDSFGNLSS	P69891	7.80	3.02E-03
YSVEFSEEPFGVIVRRQLD	P10253	7.80	3.67E-02
FGGPGTASRPSSSRSY	P08670	7.74	6.89E-03
VARVWSLMRFLIKGS	Q5XKP0	7.17	3.71E-02
FDDVVGETVGKTD	P27816	7.15	1.04E-03
VDDMPNALSALSDLHAH	P69905	7.06	2.42E-04
GQNPTNAEVLKV	P60660	6.88	7.90E-05
STAAVASDRVSIHSL	Q9GZM7	6.58	1.42E-02
NFRGGLGGG	P05787	6.28	4.23E-02
FGDNLVTRSY	P02545	6.26	1.23E-02
HFDLSHGSAQVKGHGKKV	P69905	6.25	6.88E-03
PHFDLSHGSAQV	P69905	6.05	4.41E-03
HVDDMPNALSALSDLHAH	P69905	5.96	9.30E-03
LDSLPLVDTHSK	P08670	5.95	2.91E-07
ARLPSGLPVSLLTL	P51884	5.91	2.25E-02
SLPLVDTHSKR	P08670	5.84	4.60E-02
YASSPGGVYATRSSA	P08670	5.74	2.78E-02
DQDEIQRLPGLA	P10619	5.63	1.40E-03
SLVGPAPWGFRL	Q96HC4	5.25	3.52E-02
PHFDLSHGSAQVKGHGKKV	P69905	4.84	3.15E-03
VKPVKVSAPRVGGKR	P83731	4.22	1.19E-02
VLSPADKTNVKAAWGKVGAHAGEYG	P69905	4.07	2.84E-02
AHAGEYGAEALERMFLSF	P69905	3.60	4.78E-02
LVVYPWTQRF	P02042	3.55	1.56E-02
VAHVDDMPNALSALSDLHAH	P69905	3.08	7.88E-03
FLSFPTTKTYFPHFDLSHG	P69905	2.92	2.78E-02
VTGVASALSSRY	P69892	2.91	3.54E-02
AHVDDMPNALSALSDLHAH	P69905	2.84	7.85E-03
VAHVDDMPNAL	P69905	2.76	1.58E-02

LMIEQNTKSPLFMGKVVNPTQK	P01009	2.72	1.47E-03
NRGASQAGMTGYGRPRQ	Q01995	2.68	1.51E-02
RVDPVNFKLL	P69905	2.54	1.27E-02
PWTQRFFDSF	P69891	2.52	1.61E-02
KDSGRDYVSQFEGSALGKQLNL	P02647	2.51	1.64E-02
MDSAGQDINLNSPNKG	O43399	2.47	3.08E-02
HVDPENFKLLGNVLVT	P69891	2.42	2.56E-02
SFPTTKTYFPHFDL	P69905	2.34	3.42E-02
LHTKGALPLDTVTF	P30040	2.31	2.33E-02
GGVYATRSSAVR	P08670	2.22	1.94E-02
GHFTEEDKATI	P69891	2.17	2.33E-03
IVDGKVVSETNDTKV	P05783	2.16	4.08E-02
FESFGDLSTPDAVMGNPKVK	P68871	2.14	3.85E-02
MIEQNTKSPLFMGKVVNPTQK	P01009	2.11	1.08E-02
WGKVGAHAGEYGAEALERMFLSF	P69905	2.04	3.58E-02
PLPGGVVPRMLITIL	P17931	2.04	3.60E-02
SQAYSSSQRVSS	P17661	1.92	2.39E-03
DALTNAVAH	P69905	1.92	4.90E-02
AWGKVGAHAGEYGAEALERMFLSF	P69905	1.91	1.28E-02
HVDPENFKLLGNVLVTVL	P69891	1.90	9.16E-03
NRGASQAGMTGYGRP	Q01995	1.85	2.13E-03
FVELGTQPATQ	P02652	1.82	9.45E-03
PVTVTRTTITTTT	Q96S97	1.81	1.96E-02
SLDKFLASVSTVL	P69905	1.77	3.15E-02
LSALEEYTKKLNTQ	P02647	1.69	3.33E-02
GNLSSASAIMGNPKVK	P69891	1.66	2.79E-02
TTEVGSVSEVKKDSSQ	O43491	1.62	6.15E-03
SQAYSSSQRVSSY	P17661	1.58	1.55E-02
SALSDLHAHKL	P69905	1.55	6.49E-03
AWGKVGAHAGEYGAEALERM	P69905	1.54	1.65E-02
ASLDKFLASVSTVL	P69905	1.52	3.89E-02
EQNTKSPLFMGKVVNPTQK	P01009	1.51	3.99E-02
LLPKKTESHHKAKGK	P0C0S8	1.49	2.29E-02
HVDDMPNALSALSDLHAHKL	P69905	1.47	3.66E-02
SSASAIMGNPKVK	P69891	1.36	3.67E-02
AHVDDMPNALSALSDLHAHKL	P69905	1.28	2.84E-02
AGKQAVSASGKWLDGIRKW	P14927	1.27	1.83E-02
TNAVAHVDDMPNALSALSDLHAHKL	P69905	1.26	3.61E-02
SDLHAHKLRVDPVNFKLLSH	P69905	1.24	3.10E-02
VVSETNDTKVLRH	P05783	1.23	3.89E-02
KLLGNVLVTVL	P69891	1.22	3.64E-02
VLSPADKTNVKA	P69905	1.20	2.59E-02
GHFTEEDKATITSL	P69891	1.18	2.17E-02
VDDMPNALSALSDLHAHKL	P69905	1.14	4.64E-03

VLSPADKTNVKAAWGKVGAHAG	P69905	1.11	5.15E-03
VAHVDDMPNALSAL	P69905	1.07	3.17E-02
AAHLPAEFTPAVHA	P69905	1.00	1.63E-02
NLDSLPLVDT	P08670	-1.13	2.18E-03
AGVANALAHKYH	P02042	-1.24	2.94E-02
VAGVANALAHKYH	P02042	-1.31	4.69E-02
SDGLAHLDNLKGTFATL	P68871	-1.39	1.80E-02
SYFVELGTQPATQ	P02652	-1.49	4.00E-02
QRIFENGYDPV	P08246	-1.49	4.51E-02
VHLTPEEKSAV	P68871	-1.50	2.99E-02
NWIDSIIQR	P08246	-1.53	3.04E-02
SALSDLHAHKLRVDPV	P69905	-1.67	1.75E-02
LKQVHPDTGI	P06899	-1.71	2.11E-02
YTKKVPQVSTPTLVEV	P02768	-1.93	1.64E-02
PVQAAYQKVVAGVANALAHKYH	P68871	-1.95	4.27E-02
FYAPELLFFAK	P02768	-2.01	4.51E-02
SSFLPWIRTTMR	P08311	-2.10	2.06E-02
FVNWIDSIIQR	P08246	-2.13	4.59E-02
TEAPLNPKANREKMTQI	P60709	-2.16	9.38E-03
SFLPWIRTTMR	P08311	-2.19	3.03E-02
MQNVINTVKGKALEV	Q9NT62	-2.24	2.62E-02
DLMAHMASKE	P04406	-2.27	4.04E-02
DIAVDGEPLGRVS	P62937	-2.29	3.23E-02
GEYKFQNALLV	P02768	-2.51	2.73E-02
EGKVLPGVDALSNI	P00558	-2.60	1.76E-02
LEQQNKILL	P08670	-2.60	1.99E-02
VGGRRARPHAWPFMV	P08246	-2.60	9.06E-03
TALAPSTMKIKIIAPPERKYSV	P60709	-2.68	4.19E-02
TFESRAQLGGPEAAK	P04792	-2.75	5.06E-03
SLSNKLTLDKLDVKGKRVVM	P00558	-2.76	3.79E-02
NRGPGLGSTQGQTI	Q00577	-2.83	1.67E-02
DVVMTQSPLSLPV	P06310	-2.84	9.23E-03
TLDGGFIYEAGLAPYKLRPV	P02788	-2.85	1.01E-02
LKQVHPDTGISSKAMGI	P06899	-3.32	5.39E-03
SRPVRNRKVVDYSQF	Q9H1E3	-3.41	4.73E-02
SSFLPWIRT	P08311	-3.52	1.06E-02
DIRPEIHENYRING	O75348	-4.95	3.07E-02
SLAALKKALAAAGYDVEK	P10412	-4.98	5.38E-03
VDDMPNALSALSDLHAHKLRVDPV	P69905	-5.16	7.59E-03
LLLHNNDQDNALEDLMARAGAS	Q9NZ09	-5.34	2.40E-03
AGLNSLEAVKRKI	P67936	-5.48	3.19E-02
TVLSAMTEEAAVAIKAMAK	Q6IS14	-5.60	2.32E-02
EYVNLPINGNGKQ	P09211	-5.62	2.82E-02
NEATGGKYVPRAVL	P68371	-5.64	1.88E-02

SGGKYVDSEGHL	Q03135	-5.82	1.34E-03
AEDMETKIKNYK	P14854	-5.85	6.77E-04
NEATGGKYVPRAIL	P07437	-5.88	2.69E-02
DLAGRDLTDYLMK	P60709	-5.91	1.38E-03
AEVEQKKKRTF	P62841	-5.91	2.81E-02
VYKVLKQVHPDTGI	P06899	-5.95	4.02E-03
SFTTRSTFST	P05783	-5.96	5.59E-04
AAGVEAAAEVAATEIK	P52272	-5.97	8.87E-03
ESEVVAGIPRTD	P07858	-6.04	3.89E-02
AQQAADKYLYVDKN	P35579	-6.10	4.26E-04
SEKAKPALEDLRQGLLPVLESFKV	P02647	-6.26	2.26E-04
SSGVSEIRHTA	P04792	-6.30	6.26E-03
FESDKLKAVKGFRNVIIGPA	P21980	-6.35	5.73E-05
NSGALTSGVHTFPAVLQS	P01857	-6.52	2.81E-04
SLDVNHFAPDELTV	P04792	-6.54	5.67E-03
SSFLPWIRTTMRS	P08311	-6.81	9.72E-03
SYNPATAKEIINVGHSFHV	P00915	-6.90	1.27E-03
ADKPDMGEIAS	P63313	-6.93	6.42E-03
AEKLGGSAVI	P23528	-7.01	4.54E-02
GDLSTPDAVMGNPKVK	P68871	-7.12	1.39E-02
TFHADICTL	P02768	-7.15	3.64E-02
NVEDAGGETLGRLLV	P69891	-7.17	4.72E-02
DDMPNALSALSDLHAHKLRVDPV	P69905	-7.22	6.08E-03
VGSQATDFGEALVRHDEF	O43852	-7.53	3.78E-03
RVVLGAHNLSRREPT	P08246	-7.54	1.89E-02
AAEVYGTERQPRTHYYAV	P02788	-7.68	3.36E-02
SEAEDASLLSFMQGYMKHA	P02656	-7.72	2.70E-02
SGDGLYEGLDWLSNQLRNQK	P84077	-7.85	4.11E-02
SSARPGGLGSSSLYGLG	P08729	-7.90	2.49E-03
NFRGGLGGGYGGASGMGGITA	P05787	-8.03	3.05E-02
AGFAGDDAPRAVFPSIVGRPRHQGV	P60709	-8.26	2.18E-03
SDKPDMAEIEKFDKSKL	P62328	-8.88	8.65E-03
QVPIEEQRG	Q04206	-9.23	1.60E-02
