# Supplementary material for: Screening and functional analysis of the differential peptides from the placenta of patients with healthy pregnancy and preeclampsia using placental peptidome
Source: Front Genet. 2022 Dec 1;13:1014836. doi: 10.3389/fgene.2022.1014836 (PMC9751626; doi:10.3389/fgene.2022.1014836)
Supplement: Supplementary file 2 [file Image1.pdf]

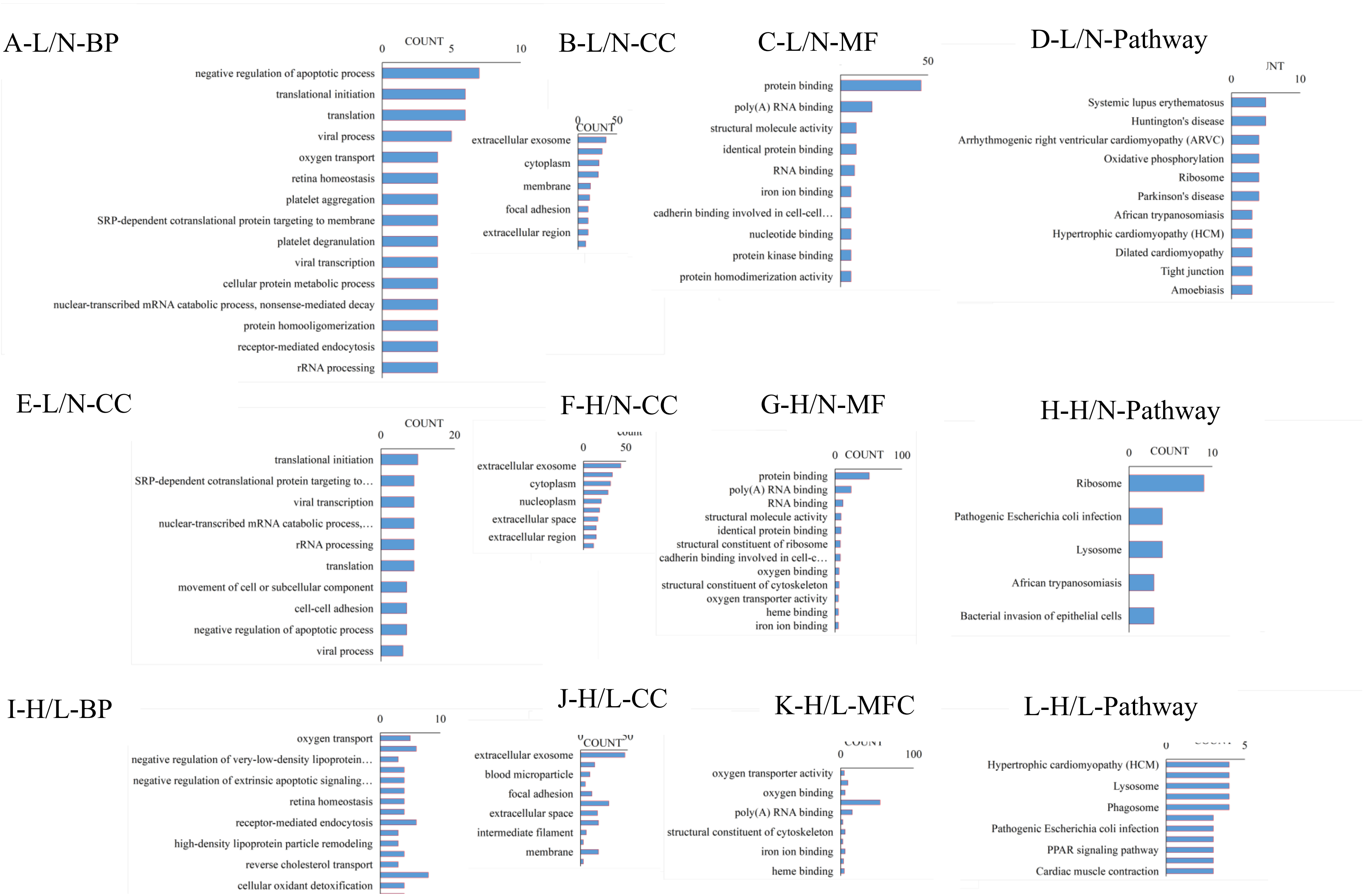

A-L/N-BP
COUNT
0
5
10
negative regulation of apoptotic process
translational initiation
translation
viral process
oxygen transport
retina homeostasis
platelet aggregation
SRP-dependent cotranslational protein targeting to membrane
platelet degranulation
viral transcription
cellular protein metabolic process
nuclear-transcribed mRNA catabolic process, nonsense-mediated decay
protein homooligomerization
receptor-mediated endocytosis
rRNA processing
B-L/N-CC
COUNT
0
50
extracellular exosome
cytoplasm
membrane
focal adhesion
extracellular region
C-L/N-MF
50
protein binding
poly(A) RNA binding
structural molecule activity
identical protein binding
RNA binding
iron ion binding
cadherin binding involved in cell-cell…
nucleotide binding
protein kinase binding
protein homodimerization activity
D-L/N-Pathway
0
10
Systemic lupus erythematosus
Huntington's disease
Arrhythmogenic right ventricular cardiomyopathy (ARVC)
Oxidative phosphorylation
Ribosome
Parkinson's disease
African trypanosomiasis
Hypertrophic cardiomyopathy (HCM)
Dilated cardiomyopathy
Tight junction
Amoebiasis
E-L/N-CC
COUNT
0
20
translational initiation
SRP-dependent cotranslational protein targeting to…
viral transcription
nuclear-transcribed mRNA catabolic process,…
rRNA processing
translation
movement of cell or subcellular component
cell-cell adhesion
negative regulation of apoptotic process
viral process
F-H/N-CC
0
50
extracellular exosome
cytoplasm
nucleoplasm
extracellular space
extracellular region
G-H/N-MF
0
COUNT
100
protein binding
poly(A) RNA binding
RNA binding
structural molecule activity
identical protein binding
structural constituent of ribosome
cadherin binding involved in cell-c…
oxygen binding
structural constituent of cytoskeleton
oxygen transporter activity
heme binding
iron ion binding
H-H/N-Pathway
0
COUNT
10
Ribosome
Pathogenic Escherichia coli infection
Lysosome
African trypanosomiasis
Bacterial invasion of epithelial cells
I-H/L-BP
0
10
oxygen transport
negative regulation of very-low-density lipoprotein…
negative regulation of extrinsic apoptotic signaling…
retina homeostasis
receptor-mediated endocytosis
high-density lipoprotein particle remodeling
reverse cholesterol transport
cellular oxidant detoxification
J-H/L-CC
COUNT
extracellular exosome
blood microparticle
focal adhesion
extracellular space
intermediate filament
membrane
K-H/L-MFC
0
100
oxygen transporter activity
oxygen binding
poly(A) RNA binding
structural constituent of cytoskeleton
iron ion binding
heme binding
L-H/L-Pathway
0
5
Hypertrophic cardiomyopathy (HCM)
Lysosome
Phagosome
Pathogenic Escherichia coli infection
PPAR signaling pathway
Cardiac muscle contraction

Figure S1 GO analyses show the function of the precursor protein of DEPs from three levels and pathway enrichment: BP, MF and CC; GO, gene ontology, BP, biological processes, CC, cell component, MF molecular function. (A, B, C, D) GO and pathway analyses the function of the precursor protein of DEPs in mild PE/control group;(E, F, G, H) GO and pathway analyses the function of the precursor protein of DEPs in severe PE/control group;(I, J, K, L) GO and pathway analyses the function of the precursor protein of DEPs in severe PE/control group; L, mild PE; H, severe PE; N, control group.
